# Primary care utilization in people who experience imprisonment in Ontario, Canada: a retrospective cohort study

**DOI:** 10.1186/s12913-018-3660-2

**Published:** 2018-11-09

**Authors:** Fiona G. Kouyoumdjian, Stephanie Y. Cheng, Kinwah Fung, Stephen Humphreys-Mahaffey, Aaron M. Orkin, Claire Kendall, Lori Kiefer, Flora I. Matheson, Samantha E. Green, Stephen W. Hwang

**Affiliations:** 1 David Braley Health Sciences Centre, McMaster University, 5th Floor, 100 Main Street West, Hamilton, Ontario L8P1H6 Canada; 2 St. Michael’s Hospital, Toronto, Canada; 3 ICES, Toronto, Canada; 4 Faculty of Medicine, McMaster University, Hamilton, Canada; 5 Schwartz/Reisman Emergency Medicine Institute, Sinai Health System, Toronto, Canada; 6 Department of Family and Community Medicine, University of Toronto, Toronto, Canada; 7 Dalla Lana School of Public Health, University of Toronto, Toronto, Canada; 8 C.T. Lamont Primary Health Care Research Group, Bruyère Research Institute, Ottawa, Canada; 9 Department of Family Medicine, University of Ottawa, Ottawa, Canada; 10 Ontario Ministry of Community Safety and Correctional Services, Toronto, Canada; 11 Centre for Criminology and Sociolegal Studies, University of Toronto, Toronto, Canada

**Keywords:** Prison, Primary care, Healthcare utilization

## Abstract

**Background:**

Access to primary care is an important determinant of health, and data are sparse on primary care utilization for people who experience imprisonment. We aimed to describe primary care utilization for persons released from prison, and to compare utilization with the general population.

**Methods:**

We linked correctional data for all persons released from provincial prison in Ontario, Canada in 2010 with health administrative data. We matched each person by age and sex with four general population controls. We compared primary care utilization rates using generalized estimating equations. We adjusted rate ratios for aggregated diagnosis groups, to explore this association independent of comorbidity. We examined the proportion of people using primary care using chi squared tests and time to first primary care visit post-release using the Kaplan-Meier method.

**Results:**

Compared to the general population controls, the prison release group had significantly increased relative rates of primary care utilization: at 6.1 (95% CI 5.9-6.2) in prison, 3.7 (95% CI 3.6-3.8) in the week post-release and between 2.4 and 2.6 in the two years after prison release. All rate ratios remained significantly increased after adjusting for comorbidity. In the month after release, however, 66.3% of women and 75.5% of men did not access primary care.

**Conclusions:**

Primary care utilization is high in prison and post-release for people who experience imprisonment in Ontario, Canada. Increased use is only partly explained by comorbidity. The majority of people do not access primary care in the month after prison release. Future research should identify reasons for increased use and interventions to improve care access for persons who are not accessing care post-release.

**Electronic supplementary material:**

The online version of this article (10.1186/s12913-018-3660-2) contains supplementary material, which is available to authorized users.

## Background

Worldwide, more than 10.3 million people are in prison at any given time [1], and an estimated 30 million people move through prisons annually [2]. On an average day, 37,864 persons in Canada and 2,217,000 persons in the USA are imprisoned in jails and prisons [1].

Given the increased morbidity and mortality experienced by this population [3], primary care in prison and after prison release offers an important opportunity to improve health [4, 5]. Further, States have obligations regarding the provision of health care to persons in prison, and to attend to aftercare at the time of prison release [6]. Comparing the health care utilization rates for people who experience imprisonment with rates for the general population is a strategy to assess health care accessibility as an indicator of health equity. For example, relatively low rates of primary care utilization would suggest worse access to care and highlight the need to facilitate primary care access to improve health status.

Studies from several countries have found increased primary care utilization for people while in prison and after release [5, 7–13]. In contrast, studies in the USA have identified relatively low use of primary care in prison and on release [14–17]. Current evidence is limited in internal and external validity, however, by small or select samples [15], the lack of general population comparator groups, [5, 8, 9, 11–17] use of self-reported data, [5, 12–17] and the age of data reported [8, 10]. Overall, there is a paucity of data on primary care use for this population, especially for the period after prison release, and no longitudinal studies have examined primary care utilization in prison and on release. In addition, we lack information on the ways in which factors such as comorbidity may contribute to increased health care utilization.

In the setting of a publicly funded universal health insurance system and a publicly funded and administered prison system, we aimed to describe the utilization of primary care for persons released from provincial prison in Ontario in 2010, and to compare primary care utilization for this group with the general population.

## Methods

### Study design and setting

We conducted a retrospective cohort study. We included all persons released from provincial correctional facilities in Ontario, Canada in 2010 as the exposed group and age- and sex-matched persons from the general population as the unexposed group. In Canada, provincial correctional facilities generally house persons who are admitted to prison prior to sentencing and who are sentenced to less than two years in prison [18]. We use the term “provincial prison” to represent all provincial correctional facilities, including jails and detention centres.

For Ontario residents (including Canadian citizens, permanent residents, Indigenous persons, and persons working full-time on a valid work permit, for whom Ontario is their primary residence), health care including hospitalizations, medically necessary surgeries, physician services including primary care, and medical tests are paid for through the public health insurance system, the Ontario Health Insurance Plan (OHIP) [19]. OHIP pays for health care provided in provincial prison and in the community, though additional health care costs are paid for in provincial prison, such as the cost of prescription medications.

In Ontario, provincial prisons are publicly funded and administered through the Ministry of Community Safety and Correctional Services (MCSCS). People in provincial prison access primary care routinely for an initial admission within weeks of admission or prior to this if medically indicated, and subsequently based on identified need for ongoing or episodic care by health care staff or through patient request. Physician employment arrangements vary across provincial prisons, but in general physicians are contracted by the ministry to provide on-site health services under OHIP. At some facilities, the contracted physician may hire additional physicians to work with them, and in a few other facilities the ministry contracts a private health service agency to recruit and contract physicians to provide services. Many physicians practice in the community as well as in a provincial prison.

### Study cohort

For this study, the MCSCS provided identifying data on all persons 18 years old or older who were released from provincial prison in 2010, including name, date of birth, sex, self-reported race, and OHIP number. The MCSCS also provided dates of admission and release and reasons for release between 2005 and 2015. The MCSCS transferred these data to ICES, an independent, non-profit organization funded by the Ontario Ministry of Health and Long-Term Care, which houses health administrative data for Ontario residents.

As described previously [20], we linked data on persons released from provincial prison with an encoded health card number (IKN) in the Registered Persons Database, which is a comprehensive database of all persons in Ontario who are eligible for coverage through OHIP [19]. To link, we used the OHIP number when provided and valid. If the OHIP number was unavailable or invalid, we used a validated method to link people deterministically or probabilistically using name and date of birth [21]. We excluded linkages that were apparently invalid (Additional file 1). We limited the sample to persons released to the community in 2010 (Additional file 1) since we were interested in access to primary care in the community post-release; we called this the prison release group.

For each person in the prison release group, we randomly selected four persons from the Registered Persons Database as general population controls from the full list of persons who had the same age and sex and were eligible for OHIP on the date of release of the person in the prison release group. We chose to match on age and sex since these factors are strongly associated with health care utilization [22, 23]. We used a ratio of 4:1 for matching to optimize statistical efficiency [24], without replacement (*i.e.* each person could be selected as a control only once).

### Variables

#### Socio-demographic information

For each person using the postal code at the time of admission to prison, we accessed data on neighbourhood income quintile (and categorized the data as missing or by quintile) and residence in rural areas/small towns (and categorized the data as missing or residence in rural area/small town or not). We used self-reported race from the MCSCS data; we maintained the category names provided by the MCSCS, *e.g.* “Aboriginal” for Indigenous persons.

#### Comorbidities

We applied previously validated algorithms [25–30] to define the proportion of persons with a prior diagnosis of the following chronic conditions: diabetes, hypertension, chronic obstructive pulmonary disease (COPD), asthma, congestive heart failure (CHF), and HIV infection. We applied definitions from the Ontario Mental Health and Addictions Scorecard and Evaluation Framework to identify persons with mood disorders, schizophrenia, substance-related disorders, and anxiety disorders [31]. To describe overall morbidity burden, we used the Johns Hopkins Adjusted Clinical Group (ACG) system [32]; for each person, we determined the number of Aggregated Diagnosis Groups (ADGs), which are 32 diagnosis clusters that indicate the burden of disease comorbidity [33]. For the mental illness diagnosis and ADGs, we used data for the two years prior to the date of initial release from provincial prison in 2010 or the corresponding date for general population controls.

#### Outcome

We defined primary care as visits to general practitioners or Family Physicians, whether in walk-in clinics or community practices. We accessed data in the OHIP database for visits for which the specialty code was “00,” which is the code for a general practitioner or Family Physician, and for which “office” was specified as the location. We excluded laboratory records and claims from nonmedical practitioners. We considered claims by the same physician for the same patient on the same day to be a single health encounter.

### Analysis

We right censored the period of follow up post-release at the earliest of death, loss of OHIP eligibility, re-admission to provincial prison (for persons released from provincial prison), or two years post-release (or corresponding date in the general population controls). We calculated person-time as the number of days in each period under study. We calculated the primary care utilization rate as the number of primary care encounters divided by person-time at risk. We calculated primary care utilization by period relative to the time in prison, *i.e.* in prison and days 0-6, 7-29, 30-89, 90-179, 180-364, and 365-730 after the initial release in 2010. We selected these periods based on prior research regarding periods of risk of adverse outcomes on release from prison, including hospitalization and death, and given our specific interest in access to primary care in the immediate post-release period as an indicator of continuity of care [20, 34–36].

We calculated rate ratios for primary care utilization for the prison release group compared to general population controls. We used generalized estimating equations with a negative binomial model, in which we controlled for correlation due to matching. We decided *a priori* to adjust for neighbourhood income quintile and rurality as potential confounders of the association between incarceration status and primary care utilization. Recognizing that comorbidity may function as an antecedent or mediating variable between imprisonment status and health care utilization, we further adjusted models for ADGs (as an indicator of comorbidity) to explore whether an association between imprisonment status and primary care utilization would persist [37].

We examined the proportion of persons in the prison release group and general population controls who accessed any primary care in each time period, and we compared the proportions between groups using chi squared tests.

We generated Kaplan-Meier curves for time to first use of primary care post-release to two years for the prison release group, stratified by sex.

For all analyses, we specified an alpha of 0.05.

We developed a protocol *a priori* (available from the corresponding author on request). We made two changes to the protocol: we included four general population controls per person in the prison release group instead of one, and we used negative binomial instead of Poisson models based on the outcome distribution.

## Results

Of 53,955 persons released from provincial prison in Ontario in 2010, we linked 52,546 (97.4%) (Additional file 1). We excluded 233 persons who had a release period of 1 day or less in 2010, 2,178 persons transferred to federal custody on release, 7 persons whose reason for release was death, and 1,267 persons whose reason for release was related to immigration, leaving 48,861 persons in the prison release group. We identified four age and sex-matched general population controls for each person in the prison release group, for a total of 195,444 general population controls.

A larger proportion of those in the prison release group were in lower neighbourhood income quintiles and were from rural areas or small towns, compared to general population controls (Table 1). The median number of ADGs was significantly greater for the prison release group compared to controls. Persons in the prison release group also had a significantly higher prevalence of all conditions examined, except for hypertension.

**Table 1:** Characteristics of persons released from provincial prison in 2010 and age- and sex-matched general population controls in Ontario, Canada

Characteristic			Prison release group, *N*=48,861	General population controls, *N*=195,444	*p* value*
Age- n (%)	Median (IQR^a^)		32 (24-43)	32 (24-43)	N/A
Sex- n (%)	Male		42,754 (87.5%)	171,016 (87.5%)	N/A
	Female		6107 (12.5%)	24,428 (12.5%)	
Self-reported race^b^- n (%)	Missing		4,499 (9.2%)	-	-
	White		28,745 (58.8%)	-	-
	Black		5,568 (11.4%)	-	-
	Aboriginal		4,954 (10.1%)	-	-
	Other		5,095 (10.4%)	-	-
Neighbourhood income quintile- n (%)	Missing		2,317 (4.7%)	1,009 (0.5%)	<.001
	1 (lowest)		18,151 (37.1%)	39,076 (20.0%)	
	2		10,481 (21.5%)	39,113 (20.0%)	
	3		7,706 (15.8%)	39,044 (20.0%)	
	4		5,923 (12.1%)	39,978 (20.5%)	
	5		4,283 (8.8%)	37,224 (19.0%)	
Rural/Small Town- n (%)	Missing		1,573 (3.2%)	164 (0.1%)	<.001
	Yes		6,339 (13.0%)	20,659 (10.6%)	
	No		40,949 (83.8%)	174,621 (89.3%)	
Time in provincial prison- median days (IQR^a^)	Admission leading to initial 2010 release		10 (3-52)	-	-
	Past five years		72 (12-230)	-	-
Time to reincarceration- median days (IQR^a^)			195 (69-490)	-	N/A
Person years of follow up (persons)	In prison^c^		6,685 (48,861)	26,738 (195,444)	N/A
	Post-release^c^	0-6 days	932 (48,861)	3,745 (195,444)	
		7-29 days	2,929 (47,870)	12,299 (195,393)	
		30-89 days	6,917 (44,939)	32,037 (195,231)	
		90-179 days	8,960 (39,328)	47,921 (194,794)	
		180-364 days	14,624 (33,538)	98,056 (194,158)	
		365-730 days	23,251 (26,055)	192,449 (193,040)	
Number of ADGs^a^- n (%)	Median (IQR)		4 (2-7)	3 (1-5)	<.001
	0-4		25,383 (51.9%)	136,412 (69.8%)	<.001
	5-9		17,395 (35.6%)	51,825 (26.5%)	
	≥10		6,083 (12.4%)	7,207 (3.7%)	
Chronic disease prevalence^d^- n (%)	Diabetes		2,341 (4.8%)	8,047 (4.1%)	<.001
	Hypertension		3,629 (7.4%)	17076 (8.7%)	<.001
	COPD^a^		2178 (4.5%)	3960 (2.0%)	<.001
	Asthma		8011 (16.4%)	26,939 (13.8%)	<.001
	CHF^a^		166 (0.34%)	507 (0.3%)	0.002
HIV^a^ infection prevalence^d^- n (%)			330 (0.7%)	343 (0.2%)	<.001
Mental disorders - n (%)prevalence^d^	Mood disorders		3,318 (6.8%)	1,521 (0.8%)	<.001
	Schizophrenia		1,909 (3.9%)	696 (0.4%)	<.001
	Anxiety disorders		3,757 (7.7%)	2,336 (1.2%)	<.001
	Substance–related disorders		8,270 (16.9%)	2,392 (1.2%)	<.001

*For chi squared or t test. ^a^*IQR* interquartile range, *ADGs*Aggregated Diagnosis Groups, *COPD* Chronic obstructive pulmonary disease, *CHF* congestive heart failure, *HIV* human immunodeficiency virus. ^b^Data on race were not available for the general population. We did not modify the category names provided by the MCSCS, *e.g.* Aboriginal. ^c^Or the corresponding dates for general population controls. ^d^Diagnosis based on health administrative data

**Fig. 1:**
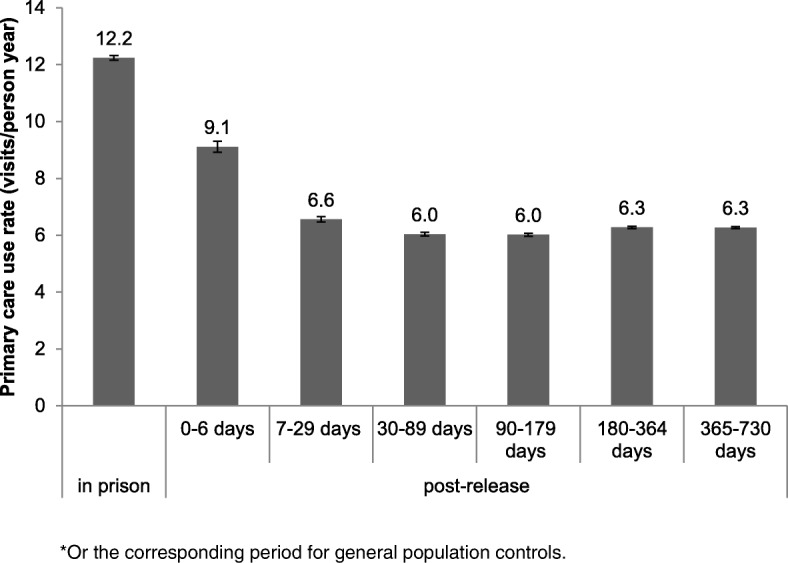
Rates of primary care utilization and 95% confidence intervals for persons released from provincial prison in 2010 in Ontario, Canada, by period relative to time in prison*

For the prison release group, primary care utilization rates were highest while in prison (Fig. 1), with a substantial decrease in use at the time of release.

**Fig. 2:**
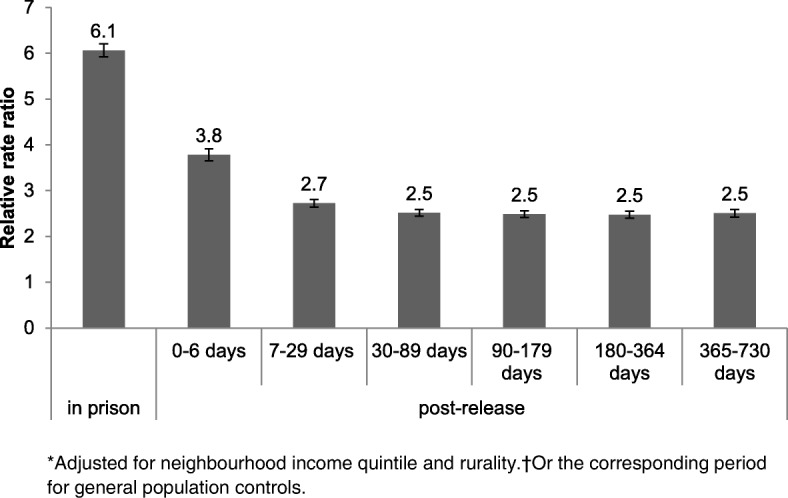
Relative rate ratio* of primary care utilization and 95% confidence intervals for persons released from provincial prison in 2010 and age- and sex-matched general population controls in Ontario, Canada, by period relative to time in prison†

The rate of health care utilization was greater for persons in the prison release group than general population controls, both in prison and in the post-release periods, as shown in Fig. 2.

As shown in Table 2, adjustment for ADGs resulted in a substantial decrease in the ratio of primary care utilization rate for the prison release group compared to general population controls. However, there remained a significant positive association across time periods between imprisonment status and primary care utilization.

**Table 2:** Unadjusted and adjusted rate ratio of primary care utilization for persons released from provincial prison in 2010 and age- and sex-matched general population controls in Ontario, Canada, by period relative to time in prison^a^

Period relative to time in prison^a^		Unadjusted rate ratio (95% CI)	Rate ratio adjusted for neighbourhood income quintile and rurality (95% CI)	Rate ratio adjusted for neighbourhood income quintile, rurality, and ADGs^b^ (95% CI)
In prison		6.1 (5.9, 6.2)	6.1 (5.9, 6.2)	3.9 (3.8, 4.0)
Post-release	0-6 days	3.7 (3.6, 3.8)	3.8 (3.7, 3.9)	2.7 (2.6, 2.8)
	7-29 days	2.6 (2.6, 2.7)	2.7 (2.6, 2.8)	1.9 (1.9, 2.0)
	30-89 days	2.4 (2.4, 2.5)	2.5 (2.4, 2.6)	1.8 (1.7, 1.9)
	90-179 days	2.4 (2.3, 2.5)	2.5 (2.4, 2.6)	1.8 (1.7, 1.9)
	180-364 days	2.4 (2.4, 2.5)	2.5 (2.4, 2.5)	1.8 (1.8, 1.9)
	365-730 days	2.5 (2.4, 2.6)	2.5 (2.4, 2.6)	1.9 (1.8, 2.0)

^a^Or the corresponding period for general population controls. ^b^*ADGs* Aggregated Diagnosis Groups

The proportion of people who accessed any primary care was significantly higher for those in the prison release group compared to general population controls for the periods in prison and post-release days 0-6, 7-29 and 30-89, as shown in Table 3. In contrast, the proportion that accessed any primary care was significantly greater for the general population compared to the prison release group for days 180-364 and 365-730 post-release.

**Table 3:** Proportion of persons released from provincial prison in 2010 and age- and sex-matched general population controls in Ontario, Canada with any primary care utilization, by period relative to time in prison^a^

Period relative to time in prison*	Prison release group		General population controls		*p* value‡
	N	Any use (%)	N	Any use (%)	
In prison^b^	48,861	40.8%	195,444	14.0%	<0.001
0-6 days post-release	48,861	13.4%	195,444	4.4%	<0.001
7-29 days post-release	47,870	19.5%	195,393	12.0%	<0.001
30-89 days post-release	44,939	28.5%	195,231	24.2%	<0.001
90-179 days post-release	39,328	33.3%	194,794	31.7%	<0.001
180-364 days post-release	33,538	43.9%	194,158	47.3%	<0.001
365-730 days post-release	26,055	58.3%	193,040	61.8%	<0.001

^a^Or the corresponding period for general population controls. Note that the period lengths vary, which limits the ability to compare the percent with any use between time periods. ^b^The median length of time in prison was 10 days (interquartile range 3 to 52 days). ‡From chi squared test of the proportion with any use in the prison release group compared to general population controls

**Fig. 3:**
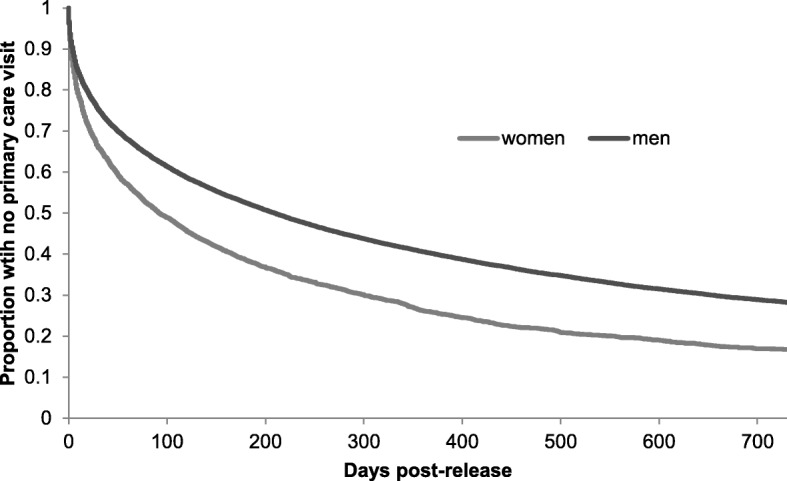
Kaplan-Meier curve of time to primary care use post-release for persons released from provincial prison in 2010 in Ontario, Canada, by sex

Figure 3 shows the time to first use of primary care after release from prison to two years. By one month after release, 66.3% of women and 75.5% of men had not yet accessed primary care and by three months after release, 50.5% of women and 62.9% of men had not yet accessed primary care. By two years after release, 16.8% of women and 28.2% of men had not accessed primary care.

## Discussion

This study identifies very high absolute and relative rates of primary care utilization in people who experience imprisonment, with over six-fold the primary care utilization rate in prison and more than twice the utilization rate post-release compared to general population controls. The associations did not change substantially after adjusting for neighbourhood income quintile and rurality, and the positive associations persisted after adjusting for comorbidity. In prison and in the months after release, a higher proportion of people in the prison release group accessed care compared to the general population controls for the same period. We note, however, that a substantial proportion of people in the prison release group did not access primary care in the months after release; more than half of women and more than 60% of men had not accessed primary care by three months post-release.

In prison, the rate of primary care utilization in our study was similar to rates in other studies, which were between 5 and 20 visits per year [7–12]. Regarding use of primary care after prison release, the proportion accessing primary care was higher in our study than in a sample of women leaving jail in New York City between 1997 to 2001, in which 47% reported primary care use in the year post-release [15], and lower than in a study of persons released from prison in Australia, in which 43% of men and 58% of women accessed care in the month post-release [5]. These differences may reflect differences in the health care system, for example the lack of a universal health insurance system may have contributed to lower primary care utilization in the New York City study. There is a paucity of available and comparable data on the proportion of persons accessing primary care in prison or on rates of primary care utilization post-release [14].

This study has limitations that may affect internal and external validity. We were not able to discern whether primary care visits in prison were for administrative reasons only, *e.g.* for ministry-required physician assessments, or whether ministry-required physician assessments were included in OHIP billings and therefore represented in health administrative data. We think it is unlikely that these issues would substantially alter our findings. We did not include primary care encounters in Community Health Centres, which provide primary care for an estimated 4% of the Ontario population [38] and about 5% of persons who experience imprisonment (data available from author on request). This exclusion would likely have led to a conservative bias in the relative rate ratios. A limitation due to the longitudinal nature of the study is that a substantial proportion of persons who were released in 2010 were not included in some follow up periods in the months after release due to right censoring as described in the analysis plan, such that by 6 months after release, only 68.6% of persons were still in the community (as per Table 3), largely due to readmission to prison. If utilization of primary care were associated with risk of readmission to prison, this could contribute to apparent differences in use over time. However, as the largest differences in rates of use occur between the time in prison and the immediate post-release period, by which time very little right censoring had occurred, we think it is unlikely that this would explain the general pattern of use by period post-release. Finally, the study results may not be generalizable to other jurisdictions, for example to jurisdictions with different healthcare systems (including those with no universal health insurance program), criminal justice systems, or prison environments, or in which patterns of morbidity and mortality differ in prison or general populations.

Various factors may contribute to the high rate of primary care utilization in this population, including increased comorbidity burden, increased injury and acute illness, and a low threshold for seeking care in prison. Data from our study and from previous reviews [3, 39] reveal high comorbidity in this population, which could lead to greater primary care use. However, even after controlling for ADGs, the positive association between imprisonment status and primary care utilization persisted. While ADGs have been shown to predict morbidity [40], mortality [33], and health care utilization, [41, 42] their validity as a comorbidity indicator has not been assessed in people who experience imprisonment; even after controlling for ADGs, there may be a residual effect of comorbidity. More complex models and more detailed data would be valuable to explore specific mechanisms in greater detail. The social and physical environment, as well as risk behaviours in prison and in the community may also increase the risk of injury or acute illness and stress in this population [8, 39, 43–46], which could contribute to primary care use. These factors would not be reflected in ADGs, which capture only current diagnoses and their sequelae. Other studies have suggested that increased primary care use in prison may reflect care seeking for issues that would be managed in the community without seeing a physician, for example through discussion with friends, family or a pharmacist [7], and that the low opportunity costs of seeing a physician in prison may encourage care use [10]. These factors may explain some of the difference in use in prison and post-release, but would not explain the increased use post-release compared to the general population.

While relative rates of health care utilization were consistently higher for the prison release group compared to general population controls, we note that only a minority of people in the prison release group accessed any primary care in the weeks after release. This is concerning given the high morbidity identified in this population in this study and others [3, 39], for which patients may need and want ongoing medical care, and given the high risk of adverse outcomes in the immediate post-release period [20, 34–36]. Of note, the increased rate of primary care utilization for the prison release group in the week after release compared to later post-release periods could reflect people appropriately accessing primary care for continuity of care for ongoing medical and social issues or to address new issues associated with the transition back to the community. However, it could also signal a lack of attention to health needs prior to release, such as providing bridging medications or prescriptions. Other types of data, such as data from medical charts and interviews, would be required to elucidate the reasons for the difference in primary care utilization rates over the post-release period.

Further research should explore primary care use in this population, including reasons for seeking care in prison and after prison release, and care experiences including access to preventive care and quality of care. This work should be done in collaboration with affected populations including people with a lived experience of imprisonment. In parallel with research, we should advance work to improve appropriate access to and acceptability of primary health care for this population. Building on the limited evidence to date [4, 13, 47–50], interventions should focus on improving access to care and quality of health care in prison and post-release.

## Conclusions

This large population-based study shows that people who experience imprisonment in Ontario, Canada have very high rates of primary care utilization compared to the general population. This association is only partly explained by increased comorbidity. Of note, a substantial proportion of people in the prison release group did not access primary care in the months after release. Research is required to understand the reasons for increased primary care utilization in this population and to support access to care for persons who are not accessing care post-release.

## Additional file

Additional file 1: Flow chart for linkage of data. (DOCX 44 kb)

## Abbreviations

ADGs: Aggregated Diagnosis Groups
CHF: Congestive heart failure
COPD: Chronic obstructive pulmonary disease
HIV: Human immunodeficiency virus
IQR: Interquartile range
MCSCS: Ministry of Community Safety and Correctional Services
MOHLTC: Ministry of Health and Long-Term Care
OHIP: Ontario Health Insurance Plan
