# Supplementary material for: Primary care utilization in people who experience imprisonment in Ontario, Canada: a retrospective cohort study
Source: BMC Health Serv Res. 2018 Nov 9;18:845. doi: 10.1186/s12913-018-3660-2 (PMC6234797; doi:10.1186/s12913-018-3660-2)
Supplement: Supplementary file 1 — Flow chart for linkage of data. (DOCX 44 kb) [file 12913_2018_3660_MOESM1_ESM.docx]

**Appendix 1. Flow chart for linkage of data**

Persons released from provincial prison in Ontario in 2010:

53,955 persons

Direct, deterministic, or probabilistic linkage with ≥1 valid IKN:
53,331 persons, 60,785 person-IKN linkages

Valid linkage:

52,546 persons, 58,363 person-IKN linkages

Excluded linkages that were likely incorrect:

- date of birth different in MCSCS and RPDB data (n=1,569)
- sex different in MCSCS and RPDB data (n=200)
- IKN matched to multiple persons (n=572)
- MCSCS data showed the person was in prison after the date of death in the RPDB (n=45)
- RPDB showed the person was OHIP-eligible after the date of death in MCSCS data (n=36)

Excluded persons not released to community in 2010:

- release period of ≤ 1 day (n=233)
- transfer to federal prison on release (n=2,178)
- died in provincial prison (n=7)
- reason for release related to immigration (n=1,267)

Prison release group:

48,861 persons
